# Cardiovascular risk and biopsychosocial interplay: Association among hypertension, anxiety, and emotional dysregulation—observational study in primary care setting for efficient self‐care

**DOI:** 10.1002/clc.24152

**Published:** 2023-09-28

**Authors:** Dina Di Giacomo, Jessica Ranieri, Federica Guerra, Eleonora Cilli, Luigi Sciarra, Silvio Romano

**Affiliations:** ^1^ Life, Health and Environmental Sciences Department University of L'Aquila L'Aquila Italy; ^2^ Di Lorenzo Clinic Avezzano Italy; ^3^ Heart Failure Clinic ASL Avezzano‐Sulmona L'Aquila Italy

**Keywords:** anxiety, biopsychological approach, cardiovascular risk, emotional dysregulation, hypertension, psychological distress

## Abstract

**Background:**

Aim of the study was to explore the relationship between emotional dimensions of hypertensive patients and the self‐care skills; we tried to draw the psychological aspects could impact the health management in hypertension analyzing the effect of emotional regulation on self‐care skills: our scope was to highlight the psychological dynamics into behavioral medicine approach.

**Methods:**

In an observational study design, patients were collected. Patients with diagnosis of hypertension were recruited in primary care setting. Inclusion criteria included patients older than 18 years, with known and medically treated primary hypertension undergoing antihypertensive medication.

**Results:**

Statistical analysis was carried out based on the data of 28 primary hypertensive patients (seven females, 21 males, mean age ± SD: 49.8 ± 7.8 years mean; clinic blood pressure: mean systolic blood pressure: 137.2 ± 13.1 mmHg, mean diastolic blood pressure: 82.1 ± 9.9 mmHg). Mean duration of hypertension in the sample was 13.1 years (±8.2 years). Correlations among the main variables of interest showed a positive and significant relationship between emotional dysregulation indexes, psychological distress, and self‐care domains: awareness resulted negatively and significantly correlated to self‐efficacy; nonacceptance, goals and impulse indexes seemed positively and significantly correlated to anxiety and depression; finally, stress was correlated positively and significantly to awareness and impulse.

**Conclusions:**

Evidencing the role of emotion dysregulation on self‐care skills and psychological outcomes, and specifically highlighting the impact of emotion dysregulation on self‐care, our findings could inform the development and implementation of psychological interventions aimed at promoting psychological well‐being and healthy behavior by focusing on the promotion of emotion regulation strategies, to reduce the risk for co‐morbidity and/or severe cardiovascular diseases.

## Graphical abstract

The study focused on the health management of the hypertensive patients over the time considering the emotional perspective on the self‐care skills. As expected, anxiety resulted a co‐morbidity in hypertension; interesting results was to detect the related association between psychological and health management factors. In particular, emotional dysregulation and psychological distress were correlated to self‐care.

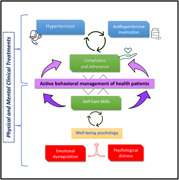

**Abbreviations**
BP: blood pressure
DASS‐21: Depression Anxiety Stress Scales 21
DBP: diastolic blood pressure
DERS: Difficulties in Emotion Regulation Scale‐20
SBP: systolic blood pressure
SC‐CII: Self‐Care of Chronic Illness Inventory

## 1 INTRODUCTION

Essential hypertension can be defined as “a rise in blood pressure of unknown cause that increases risk for cerebral, cardiac, and renal events.”^1^ According to several studies, hypertension is the main risk factor for the development of stroke, coronary heart disease, congestive heart failure and chronic kidney disease.^2^, ^3^, ^4^, ^5^, ^6^ Moreover, among patients with cardiovascular disease, depression and/or anxiety are very common, with a prevalence ranging from 15% to 50%. Affective and emotional disorders are associated with increased risk for incident cardiovascular events, rehospitalization, all‐cause and cardiovascular mortality both in patients with overt cardiac disease^7^; unhealthy lifestyle,^8^ inadequate adherence to medical prescriptions, themselves associated with emotional disorders are considered risk factors for reduced Quality of Life as well physical condition.^9^, ^10^ Negative emotions as anger and hostility might be associated with harmful effects greater in cardiovascular patients than healthy population^11^; more, frequent episodes of anger and hostility traits might accelerate recurrence of events in cardiovascular patients.^12^ Behavioral epidemiology highlighted the cardiac risk for negative emotions; based on historical perspective, the individuals with history of frequent anger expression were at high risk of developing essential hypertension. Although lacking for empirical evidence for this mechanism, the belief still persists because the idea that keeping in angry feelings is related to “hyper‐tension” is intuitively compelling.^13^ Cognitive, behavioral and cognitive‐behavioral interventions associated with pharmacological treatments of anger/hostility have the potential to reduce anger and its health‐damage effects.^14^

Anxiety is more common in patients with hypertension, and these two conditions frequently coexist. Recently, some studies focused on determining etiology in patients with comorbid hypertension and anxiety^7^ highlighting a positive association between them. The relationship between blood pressure control and central nervous system (CNS) function in the development of essential hypertension is complex and multidimensional. Emotional dysregulation is related to levels of resting blood pressure, total peripheral resistance, and other indices of hemodynamic function in individuals who are significantly elevated risk for cardiovascular disease; considering that, the links among CNS regulation of emotions, neural control of circulation and hypertension development appeared relevant.^15^ According to lately Schaare's study,^15^ even the multidimensional pathways between blood pressure and mental health are still to deeply investigate, the mechanism between subjective experience, emotional processing, and pain involves the regulatory baroreflex system and altered sensory processing.

Furthermore, hypertension and anxiety are associated with lower treatment compliance, lower levels of daily functioning, lower health‐related quality of life, and healthcare‐related costs.^16^ According to that, health management and monitoring seemed relevant for the efficacy of medical/pharmacological treatments^2^; more, hypertension is associated to greater risk of cardiovascular disease‐related mortality and common risk factors and potential mechanisms of comorbid might be the co‐occurrence of hypertension and anxiety.^4^

The co‐occurrence of hypertension and anxiety suggests the existence of mutual risk factors. Individual risk factors, lifestyle choices, and environmental risk factors are the primary risk factors associated with comorbid hypertension.^7^ Even psychosomatic factors could influence hypertensive medical treatment: psychological distress could impact the medical practice as well the no‐awareness for the patients regarding the own hypertension condition, and favoring the failing the efficacy of antihypertensive drugs.^17^, ^18^

Considering the literature, mental health disorders in hypertension could be influencing the Quality of Life of patients making mental health the emerging clinical topic: although hypertension could be viewed in itself as a biomedical problem, patients experience with the demands of living as hypertensives resulted in mental health problems. This implies the link between biomedical problems and the development of psychological disorders^11^, ^19^; the scenario of biopsychosocial approach for clinical practice is going to reinforced for the patient care toward to adherence medications and efficient setting care.^2^

Taking into account the literature, age, sex, smoking, alcohol abuse, obesity, lead exposure contribute to the development of comorbid hypertension and anxiety. Preventive measures such as risk factor screening and early health management can enhance patient well‐being. More importantly, the specific mechanisms involved in comorbid hypertension and anxiety require further investigations: in particular, the emotional dimensions related to the anxiety might be analyzed in depth, drawing the complexity of behavior adaptation toward medical care.

However, it remains unclear the influence of hypertension development and mental health. The investigations regarding the interplay between psychological mechanisms and behavioral adaptation in cardiovascular risk for efficient medical treatments is an emerging research topic; the impact is related to the implementation consistently of the cardiologic clinical practice in primary care setting enhancing the efficacy of medication over the time.

Taking into account that, our study wanted to explore the relationship between emotional dimensions of hypertensive patients and the self‐care skills: we aimed to analyze the impact emotional regulation in the active care of patient for efficient adherence medication.

Our research hypothesis was the patients with hypertension and psychological disorders could be less active in the clinical process; the emotional dysregulation could negatively affect the behavioral adjustment to tailored pharmacological treatments. In our study, we tried to draw the psychological aspects could impact the health management in hypertension analyzing the effect of emotional regulation on self‐care skills: our scope was to highlight the psychological dynamics into behavioral medicine approach.

## 2 MATERIALS AND METHODS

### 2.1 Ethic approval

The study was conducted according to the guidelines of the Declaration of Helsinki and approved by the Institutional Review Board of the University of L'Aquila, Italy (Prot. No.37590/2021).

### 2.2 Patients

In an observational study design, patients were collected. Patients with diagnosis of hypertension were recruited in primary care setting of Cardiology Unit of ASL1 Abruzzo (Italy).

Inclusion criteria included patients older than 18 years, with known and medically treated primary hypertension undergoing antihypertensive medication.

Exclusion criteria were (a) secondary hypertension, (b) the presence of ICD‐10 depressive or bipolar disorders or antidepressant/mood stabilizer treatment at the time of the study.

### 2.3 Measurements

#### 2.3.1 Clinical measures

Patients with diagnosis of hypertension were measured by the systolic blood pressure (SBP) and diastolic blood pressure (DBP). Hypertension was defined according to the seventh report of the Joint National Committee on Prevention, Detection, Evaluation, and Treatment of High Blood Pressure (SBP/DBP ≥ 140/90 mmHg or use of antihypertensive medication).

#### 2.3.2 Psychological measures

##### Depression Anxiety Stress Scales 21 (DASS‐21)

The DASS‐21 is a self‐administered questionnaire that measures the degree of severity of the core symptoms of depression, anxiety, and stress. It is composed of 21 questions with responses on a four‐point Likert‐type scale.

##### Difficulties in Emotion Regulation Scale‐20 (DERS)^20^

It is an instrument measuring emotion regulation issue. The 20 items self‐report scale asks respondents how they relate to their emotions to produce scores on the following subscales: (1) Nonacceptance of emotional responses; (2) Difficulty engaging in goal‐directed behavior; (3) Impulse control difficulties; (4) Lack of emotional awareness; (5) Limited access to emotion regulation strategies. This scale measures an integrative conceptualization of emotion regulation as involving not just the modulation of emotional arousal, but also the awareness, understanding, and acceptance of emotions, and the ability to act in desired ways regardless of emotional state.

##### Self‐Care of Chronic Illness Inventory (SC‐CII)^21^

The SC‐CII is a 20‐item self‐report questionnaire that assesses the self‐care process followed by individuals with a variety of chronic conditions. It measures self‐care, defined as a naturalistic decision‐making process involving health‐promoting practices and illness management, that includes self‐care maintenance, self‐care monitoring, self‐care management, and self‐efficacy. Self‐care maintenance primarily reflects health‐promoting and maintenance behaviors such as exercise and taking medication as prescribed. Self‐care monitoring involves checking oneself for changes in signs and symptoms. Self‐care management reflects the response to changes in signs or symptoms, if and when they occur (e.g., adjusting diet or medication based on detection and interpretation of symptoms). Self‐care management, maintenance, and monitoring dimension is then measured by the relevance scale, where the score is standardized from 0 to 100. Scores >70 indicated adequate levels of self‐care. Self‐efficacy refers to an individual's belief in their capacity to execute behaviors necessary to produce specific performance attainments.

### 2.4 Procedure

Medical staff in the Cardiology Unit identified eligible patients, who were then enrolled during a check‐up by medical protocol for the management of pharmacological treatment. Participants were outpatients recruited from the cardiology clinic during scheduled follow‐up. Written informed consent was obtained from all participants at the time of enrolment. Trained clinical psychologists (blinded to the objectives of the study) conducted the psychological evaluations in a quiet room. The evaluations lasted for 20 minutes. Participants completed the measures during their scheduled follow‐up. Data were collected anonymously.

## 3 RESULTS

### 3.1 Baseline characteristics of the sample

Statistical analysis was carried out based on the data of 28 primary hypertensive patients (seven females, 21 males, mean age ± SD: 49.8 ± 7.8 years mean; clinic blood pressure: mean SBP: 137.2 ± 13.1 mmHg, mean diastolic blood pressure: 82.1 ± 9.9 mmHg). Mean duration of hypertension in the sample was 13.1 years (±8.2 years). Most common chronic disorders were musculoskeletal disorders (38.1%), diabetes mellitus (22.8%), and gastrointestinal diseases (21.5%).

First, we wanted to verify the psychological dimensions into the One sample (*t*‐Student) test; statistical analyses (Table 1) showed significant difference (*p* = < .001) into examined psychological dimensions.

**Table 1:** One sample *t*‐student test statistical analysis on the psychological testing.

	Statistic	*df*	*p* Value	Mean difference	Effect size Cohen's d
DASS‐21					
Depression	5.06	27.0	<.001	6.71	0.95
Anxiety	5.16	27.0	<.001	8.50	0.97
Stress	8.08	27.0	<.001	16.07	1.52
DERS‐20					
Nonacceptance	11.10	27.0	<.001	8.64	2.09
Awareness	16.34	27.0	<.001	9.32	3.08
Goals	11.90	27.0	<.001	8.11	2.25
Clarity	10.76	27.0	<.001	5.07	2.03
Impulse	10.68	27.0	<.001	6.89	2.01
Self‐care					
Maintenance	34.4	27.0	<.001	28.1	2.50
Monitoring	22.7	27.0	<.001	19.7	4.28
Management	18.9	27.0	<.001	19.2	3.56
Self‐efficacy	28.6	27.0	<.001	39.4	5.41

Abbreviations: DASS‐21, Depression, Anxiety, Stress Scale; DERS‐20, Difficulties in Emotion Regulation Scale; *df*, difference; p, significance.

### 3.2 Correlation between psychological distress, emotional regulation, and self‐care skills

Correlations among the main variables of interest showed a positive and significant relationship between DERS indexes and psychological distress (DASS‐21 indexes) and self‐care domains: awareness resulted negatively and significantly correlated to self‐efficacy; nonacceptance, goals, and impulse indexes seemed positively and significantly correlated to anxiety and depression; finally, stress was correlated positively and significantly to awareness and impulse.

Pearson's coefficients are shown in Table 2.

**Table 2:** Correlation matrix among psychological distress (DASS), emotional dysregulation (DERS), and self‐care.

	DERS	DERS	DERS	DERS	DERS
	Nonacceptance	Awareness	Goals	Clarity	Impulse
Self‐care (maintenance)					
Pearson's *r*	−.077	.111	−.115	.013	−.115
*p* Value	.698	.575	.561	.948	.561
Self‐care (monitoring)					
Pearson's *r*	.043	−.345	−.195	.060	.045
*p* Value	.830	.072	.321	.761	.820
Self‐care (management)					
Pearson's *r*	.069	.025	.117	.219	.060
*p* Value	.729	.899	.553	.262	.763
Self‐efficacy					
Pearson's *r*	.000	−.417*	−.148	−.067	−.129
*p* Value	.999	.027	.451	.735	.513
DASS—Anxiety					
Pearson's *r*	.679***	.224	.432*	.199	.514**
*p* Value	<.001	.251	.022	.309	.005
DASS—Stress					
Pearson's *r*	.515**	.349	.328	.262	.398*
*p* Value	.005	.069	.088	.177	.036
DASS—Depression					
Pearson's *r*	.488**	.202	.506**	.162	.575**
*p* Value	.008	.303	.006	.410	.001

Abbreviations: DASS, Depression, Anxiety, Stress Scale; DERS, Difficulties in Emotion Regulation Scale.

* *p* < .05

** *p* < .01

*** *p* < .001.

### 3.3 Path model

**Figure 1:**
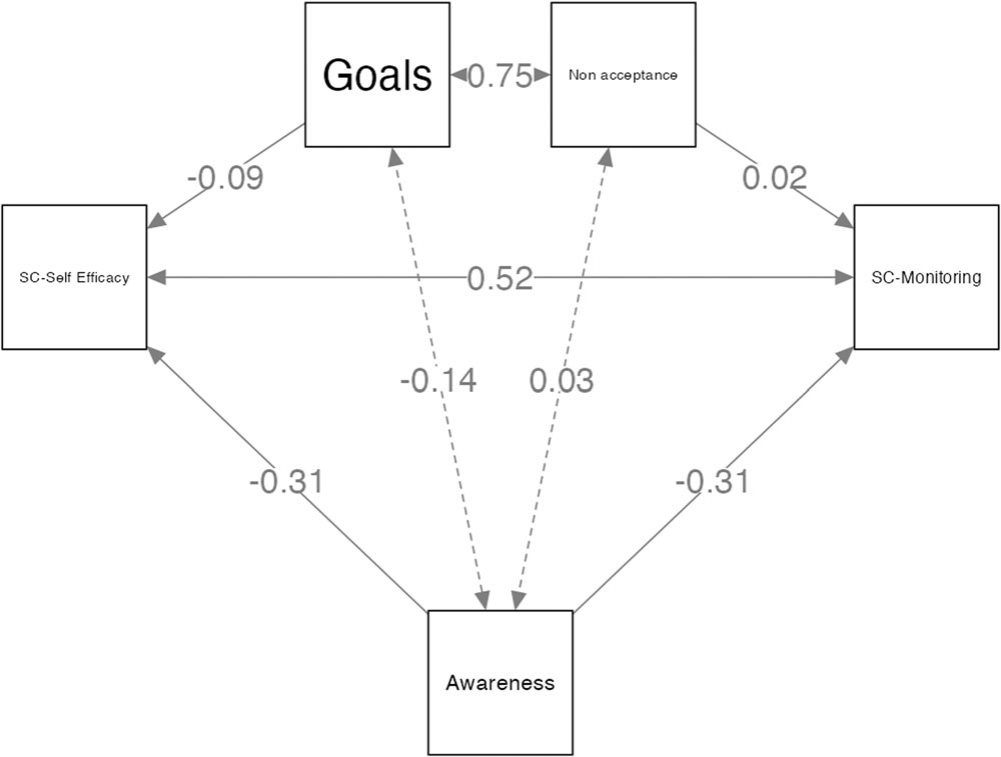
The path for the significant relationship between nonacceptance, awareness, and goals (DERS indexes) and monitoring and self‐efficacy abilities (self‐care indexes). Legend: Beta values were reported. DERS, Difficulties in Emotion Regulation Scale.

The path model was presented in Figure 1. We tested a model in which DERS was hypothesized to be a significant predictor of self‐care indexes and anxiety; DERS indexes were supposed to be predictors of self‐care skills.

The path model showed good indices of fit: *χ* ^2^ (25.4; *p* = .003), CFI (1.00), TLI (1.000), SRMR (0.000), RMSEA (0.000). Specially, nonacceptance significantly predicted the monitoring ability (b = 0.50; SE: 0.21; *p* = <.045; 95% BC‐CI [0.009–0.83]) as well, the goals significantly predicted, monitoring (b = −3.18; SE: 0.22; *p* = <.001; 95% BC‐CI [−1.131‐0.26]); finally the awareness significantly precited the self‐efficacy (b = −0.37; SE: 0.31; *p* = <.02; 95% BC‐CI [−1.300 to −0.06]). Finally, DERS indexes did not significantly predict the management and maintenance ability of self‐care.

## 4 DISCUSSION

The study focused on the health management of the hypertensive patients over the time considering the emotional perspective on the self‐care skills. As expected, we found significant associations between all the study variables. As expected, anxiety resulted a co‐morbidity in hypertension; interesting results was to detect the related association between psychological and health management factors. In particular, emotional dysregulation and psychological distress were correlated to self‐care. More, nonacceptance, goals and awareness dimensions resulted predictors for monitoring and self‐efficacy skills. Our finding is interesting for clinical practice: hypertensive patients are required to be active in the management of own health and symptoms in daily living; the development of mental health disorders related to difficulties to regulation of emotions leads to significant skills deficits for behavioral health and well‐being; taking into account our research, the balancing of emotional dimensions and physical needs should be managed by tailored actions and strategic therapeutic advantage.

Our finding was in line with preview studies^2^, ^4^, ^5^, ^6^ and strongly highlighted the impact of psychological and behavioral interplay in cardiovascular risk. Cardiac disease could be affected in the first time of primary care to the low adherence to the medication drawing and alarming level for self‐management of health in long time.^22^, ^23^ Emotional regulation appeared the mechanisms to take care in clinical setting for adherence to medication.^11^, ^24^ According to the literature, our findings deal with the hypertension disease by biopsychological perspective evidencing the interplay between the negative health behavioral in the monitoring and perceived self‐efficacy to manage the medication and daily habits. Depression and anxiety dimensions could affect the adherence to therapeutic advices: mood disorders, psychological stress are related to blood pressure adjustment.^11^

In our opinion, physical and mental clinical treatments should be planned and tailored for individuals features to favor the adherence medication and better active behavioral management of health patients: pharmacological therapy could be addressed by identifying the emotional characteristics, subjective emotional ability, and the psychological process of patients who are or may be at risk of nonpersistence.

Such evidence offers important clinical implications. First of all, our work exceeds the limits of previous research by exploring the link between emotion dysregulation and health management in term of self‐care skills in addition to the psychological ones, which have been already studied.

Evidencing the role of emotion dysregulation on self‐care skills and psychological outcomes, and specifically highlighting the impact of emotion dysregulation on self‐care, our findings could inform the development and implementation of psychological interventions aimed at promoting psychological well‐being and healthy behavior by focusing on the promotion of emotion regulation strategies, to reduce the risk for co‐morbidity and/or severe cardiovascular diseases.

Limitations of the study are small simple size and limited biomarkers. Future research should cover additional constructs, involve other measurement strategies, and test the same model in different subgroups (i.e., different disease timing and medications).

## AUTHOR CONTRIBUTIONS

Dina Di Giacomo conceptualized the study. Jessica Ranieri and Federica Guerra collected and analyzed the data. Eleonora Cilli provided technical assistance with recruiting participants and conducting the survey. Luigi Sciarra supervised and analyzed data. Silvio Romano supervised. All authors reviewed contributed to write and review of the manuscript.

## Data Availability

The data sets generated and analyzed during the current study are not publicly available due to limitations of ethical approval involving the patient data and anonymity but are available from the corresponding author on reasonable request.
